# A Study of Brain Neuronal and Functional Complexities Estimated Using Multiscale Entropy in Healthy Young Adults

**DOI:** 10.3390/e21100995

**Published:** 2019-10-12

**Authors:** Sreevalsan S. Menon, K. Krishnamurthy

**Affiliations:** Department of Mechanical and Aerospace Engineering, Missouri University of Science and Technology, Rolla, MO 65409, USA; sm2hm@mst.edu

**Keywords:** brain complexity, dynamic functional connectivity, edge complexity, fluid intelligence, multiscale entropy, node complexity, resting-state functional magnetic resonance imaging, sample entropy

## Abstract

Brain complexity estimated using sample entropy and multiscale entropy (MSE) has recently gained much attention to compare brain function between diseased or neurologically impaired groups and healthy control groups. Using resting-state functional magnetic resonance imaging (rfMRI) blood oxygen-level dependent (BOLD) signals in a large cohort (n = 967) of healthy young adults, the present study maps neuronal and functional complexities estimated by using MSE of BOLD signals and BOLD phase coherence connectivity, respectively, at various levels of the brain’s organization. The functional complexity explores patterns in a higher dimension than neuronal complexity and may better discern changes in brain functioning. The leave-one-subject-out cross-validation method is used to predict fluid intelligence using neuronal and functional complexity MSE values as features. While a wide range of scales was selected with neuronal complexity, only the first three scales were selected with functional complexity. Fewer scales are advantageous as they preclude the need for long BOLD signals to calculate good estimates of MSE. The presented results corroborate with previous findings and provide a baseline for other studies exploring the use of MSE to examine changes in brain function related to aging, diseases, and clinical disorders.

## 1. Introduction

Fluctuations in the resting-state functional magnetic resonance imaging (rfMRI) blood oxygen-level dependent (BOLD) signals have recently received considerable attention for their use in studies of functional brain networks and discovering “neuromarkers” for brain function in diseases and clinical disorders [1]. Functional connectivity (FC) describes the correlation of two time series from different regions of the brain, which are also referred to as nodes, and similarities among the time series from a set of nodes have been used to identify resting-state networks (RSNs). In addition, these functional networks have been associated with different cognitive functions [2]. The connections between the nodes are referred to as edges, and a matrix of all pair-wise edge strengths is used to denote the FC. This matrix is symmetric with the rows and columns representing the nodes and edges, respectively.

In the past, many studies made the simplifying assumption that the correlation or functional connection between different regions of the brain is static [3]. However, dynamic functional connectivity (dFC) more accurately reflects non-stationary brain activity and is, therefore, receiving increased attention [4,5]. A common approach to find the dFC is to use a sliding window, the length of which determines the final number of time points, of the BOLD time series data to determine repeated states by using a clustering algorithm [6]. Another approach is to use the BOLD phase coherence connectivity (PCC) [7,8,9]. Dynamic functional connectivity has been explored for its ability to identify intrinsic individual brain connectivity patterns [10,11], to study age-related cognitive changes [9], and to classify brain disorders [12], to cite a few examples.

The variability and complexity of the brain’s neural signals are two quantitative measures that can be used to assess the health of a brain [13]. These measures indicate the ability of the brain to react to uncertainty and adapt to and function in a dynamic environment. Higher variability is associated with better behavioral performance, as it allows for the formation of functional networks and probes various functional configurations. Higher complexity, on the other hand, is associated with a higher information processing capacity. Healthy systems exhibit chaotic and complex behaviors; a loss of this complexity or transition to less complicated, predictable behaviors is an indication of disorders or impairments in fixed-point attractor systems and vice versa in oscillatory systems [14,15,16].

Several entropy measures have been used to study brain complexity using fMRI data. Approximate entropy [17] and sample entropy (SampEn) [18] are two such measures. Both measures estimate Kolmogorov entropy [19], which is the rate of generation of new information, and are attractive because of their immunity to low-level noise, robustness to occasional large or small artifacts, the ability to perform even with missing data, and preclude the requirement of a large number of data points. However, SampEn is advantageous because it is less dependent on the time series length being in the range 10${}^{m}$–20${}^{m}$ (*m* being the pattern length) and displays relative consistency compared to approximate entropy. SampEn is the natural logarithm of the conditional probability that a pattern length of *m* points will repeat itself, without including self matches, for m+1 points within a tolerance of *r* in a time series of length *N* [18]. Entropy is a measure of randomness and predictability of a stochastic process and, in general, increases with greater randomness. Therefore, higher SampEn values mean that the system has more complexity.

One of the issues in calculating SampEn is selecting the appropriate values for *m* and *r*. Choosing a large *m* and small *r* will result in fewer patterns, whereas a small *m* and large *r* will result in more pattern matches. These two extremes result in higher statistical variations in calculating SampEn and the reduced ability of SampEn to model temporal dynamics, respectively. It is also possible to fail in estimating SampEn for some combinations of *m* and *r* because no matching patterns can be found, and these combinations must be avoided. Adopting the strategy of [20], a systematic approach was presented in [21] to overcome limitations in previous studies wherein parameters are selected in an ad hoc fashion or those resulting in the maximum difference in population groups. Parameters are selected to keep the relative error of SampEn less than 0.1, which corresponds to about 10% of the coefficient of variation value in SampEn estimates. The relative error is estimated from the mean and standard deviation of SampEn of cerebrospinal fluid (CSF) signals, which contain minimal physiologic information (see Section 4.3 for details). Parameters that minimized this relative error are then used to calculate SampEn in BOLD signals in gray matter regions.

To more effectively model complex temporal fluctuations, multiscale entropy (MSE) was proposed to estimate dynamic complexity in a time series by considering different time scales [22]. In this method, multiple “coarse-grained” time series of length (N/l), where *l* is the scale factor, are first formed by averaging consecutive, non-overlapping data points of increasing length. Then the SampEn of each coarse-grained time series is calculated. Because complexity is evaluated over different time scales (high frequencies at fine scales (i.e., low scale factors) and low frequencies at coarse scales (i.e., high scale factors)), MSE can better identify frequency-dependent neuropathophysiological processes in different brain regions.

MSE has been used to study changes in brain complexity that are related to aging [16,23,24], Alzheimer’s disease [25,26], and schizophrenia [27,28]. SampEn was used to measure complexity within dFC in [28,29], the only two studies where SampEn was calculated using dFC between nodes and are therefore different from the other studies. However, the entropy of the dFCs was averaged to find the SampEn of nodes, RSNs, and whole brain. Patients showed a significantly higher SampEn than the controls at the whole-brain level, in two RSNs (visual recognition and auditory networks), and three nodes (right middle occipital gyrus, right inferior occipital gyrus and left superior occipital gyrus) [28]. Although the whole brain and RSNs were not correlated to the clinical variables, the three nodes did show a strong positive correlation. The results in [28,29] motivate further studies on the use of MSE of dFC, particularly at the edge level without any averaging, to develop new approaches that more effectively utilize the spatiotemporal fluctuations in the brain activity.

Using publically available rfMRI data of about 1200 healthy young adults from the Human Connectome Project (HCP) S1200 release [30], brain complexity estimated using SampEn over multiple scales is studied here. One of the distinguishing features of the HCP is that rfMRI data were collected in four 15-min sessions over two consecutive days, providing a large time series data set (4800 volumes) to more accurately estimate complexity and study variations of SampEn over a wide range of scale factors. The long rfMRI time series data of a large cohort is exploited here to map the brain complexity across scales at four different levels:

1. Edge level—edge complexity is estimated by the MSE of each edge, calculated from its dFC time series data (eMSE) that is obtained using BOLD PCC.
2. Node level—node complexity is estimated by the MSE of each node, calculated from its BOLD time series data (nMSE) or calculated as the mean eMSE of all the edges connected to the node (edge-based nMSE).
3. Network level—network complexity is estimated by the mean nMSE or mean edge-based nMSE of all the nodes in the RSN.
4. Whole-brain, consisting of only the cortex and subcortical gray matter, level—whole-brain complexity is estimated by the mean nMSE of all the nodes or mean eMSE of all the edges in the brain.

By virtue of using the dFC edge time series, eMSE and edge-based nMSE are measures of functional complexity. On the other hand, nMSE is a measure of neuronal complexity because it is estimated from the BOLD time series. Past studies have focused mostly on neuronal complexity, exploring patterns at the node level and/or RSN level. Functional complexity explores patterns in a dimension higher than the node level, and the utility of functional complexity has not yet been fully explored.

Fluid intelligence [31,32], which is the capacity to identify patterns and solve problems independent of previously acquired knowledge, is known to be involved in individual differences [33]. The eMSE, nMSE, and edge-based nMSE values are used as features to predict fluid intelligence by using the linear-kernel support vector regression (SVR) leave-one-subject-out cross-validation scheme. The prediction accuracy results obtained using eMSE, nMSE, and edge-based nMSE features will help to better understand the use of brain complexity measures as a complementary tool to the traditional functional connectivity analysis. Moreover, the results of this study involving healthy young adults will serve as a baseline for better identification of changes in brain function due to age, diseases, and clinical disorders.

## 2. Results

### 2.1. Relative Error of SampEn

**Figure 1:**
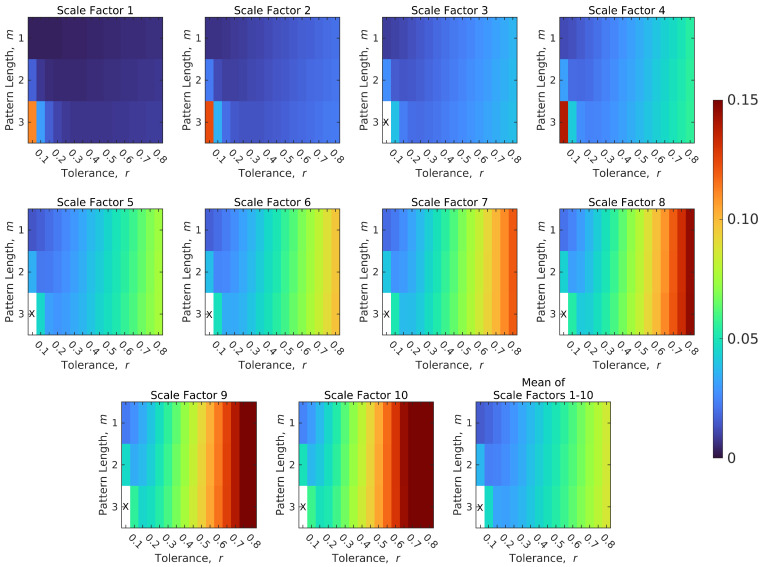
Median relative error of SampEn in CSF signals for scale factors 1–10. The color bar on the right displays the mapping of the median relative error to the color map. An “X” is used to denote failure to find a SampEn value.

Figure 1 shows a color map of the median relative error of SampEn of CSF signals across all subjects. The median relative error was calculated for 48 different combinations of pattern lengths (*m* = 1, 2, and 3) and tolerance values (*r* = 0.05 to 0.8 in increments of 0.05). These 48 error values are mapped to a color and then shown in a 3 × 16 grid of pattern lengths and tolerance values. Several expected trends can be clearly seen when the MSE parameters are varied. At fine scales, the relative error was low, and this error increased with coarser scales. For pattern lengths m=2 and m=3, the relative error for low and high tolerance values was higher compared to other values, with a “skewed U-pattern” toward the lower end. An increase in the parameter length increased the relative error in SampEn estimates. The combinations when there were failures in estimating SampEn, which occurred only when m=3, are shown with an “X”. The tolerance had to be increased with higher pattern lengths to avoid failures. In general, failures occurred when the tolerance was low and the pattern length was high, and the relative error increased with an increase in the scales.

The lowest mean relative errors across all scales for each parameter length were calculated as 0.016 (m=1 and r=0.05), 0.024 (m=2 and r=0.1), and 0.032 (m=3 and r=0.2), respectively. Instead of picking the case with the lowest relative error, the results are presented in Section 3 for the case m=3 and r=0.2, because m=3 is a stronger test of repeating patterns compared to m=1 and m=2. As previously noted, relative errors of SampEn of CSF signals were used as estimates of the relative errors of BOLD signals and BOLD PCC.

### 2.2. Brain Complexity Across Different Levels

**Figure 2:**
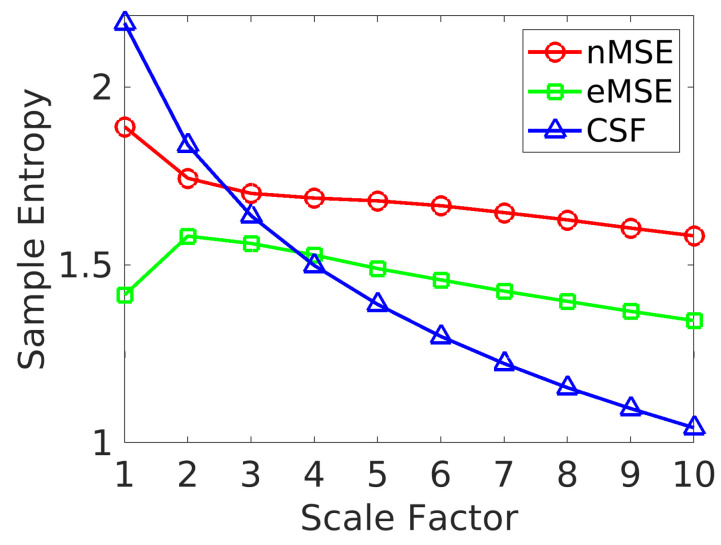
Group mean SampEn of whole brain (gray matter) and cerebrospinal fluid (CSF) signals across scale factors.

Figure 2 shows variations in the group mean (i.e., average over all subjects) SampEn across scales for the whole brain, considering only the cortex and subcortical gray matter, as previously defined. All three SampEn values decreased at coarse scales, but with different trends. The exponential decrease in CSF SampEn is similar to that of white noise [34]. There was an initial increase in eMSE, followed by a decreasing trend that also occurred in nMSE. In addition, the decrease in the slope of nMSE and eMSE was less than CSF SampEn, and the overall brain complexity estimated by nMSE was higher than eMSE for all scales. The two-tailed paired t-test for the worst case is $t_{966}=40.9057$, $p<5\times 10^{-213}$ (uncorrected). The higher nMSE and eMSE values compared to CSF SampEn at coarse scales clearly delineate neuronal signals from non-neuronal signals.

**Figure 3:**
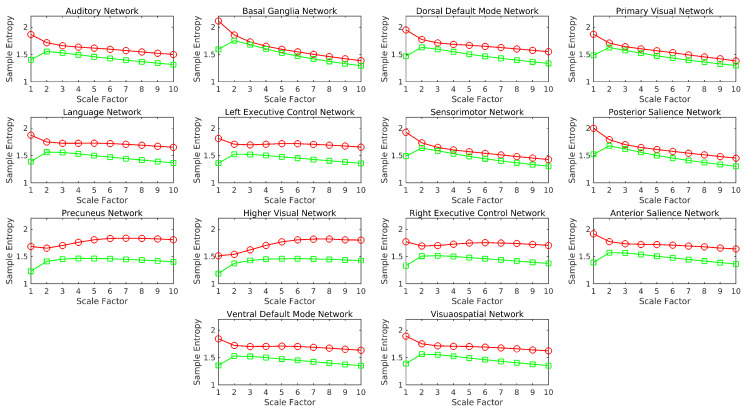
Group mean SampEn across scale factors of resting-state networks (RSNs). Red circles and line—nMSE, green squares and line—edge-based nMSE.

**Figure 4:**
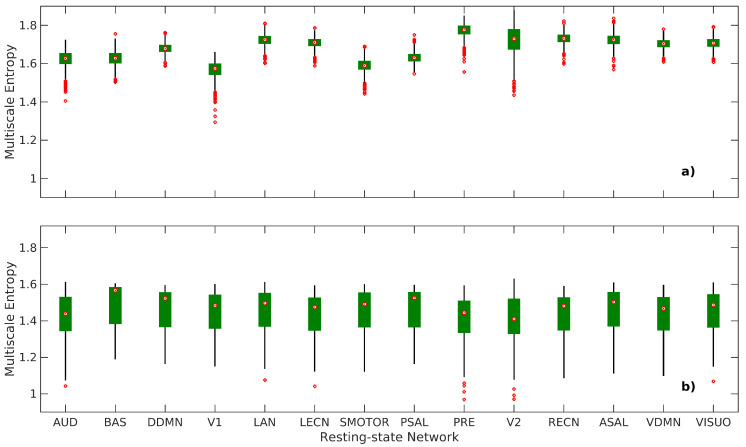
Box plots of the average MSE values of scale factors 1–10 for each RSN. (**a**) mean nMSE; and (**b**) mean edge-based nMSE. On each box, the central mark shows the median, bottom and top edges show the 25th and 75th percentiles, respectively, and the whiskers extend to the most extreme data points that are not considered as outliers, which are defined as 1.5 times the interquartile range away from the top or bottom of the box. AUD-auditory, BAS-basal ganglia, DDMN-dorsal default mode network, V1-primary visual, LAN-language, LECN-left executive control network, SMOTOR-sensorimotor, PSAL-posterior salience, PRE-precuneus, V2-higher visual, RECN-right executive control network, ASAL-anterior salience, VDMN-ventral default mode network, VISUO-visuospatial.

Figure 3 and Figure 4 show the network-level results. Figure 3 shows variations in the group mean SampEn in RSNs across the scales. Except for the precuneus, higher visual, and right executive control network (RECN), nMSE shows a decreasing trend similar to that seen in the whole brain. In the case of edge-based nMSE, all networks (with the exception of the precuneus and higher visual) show a trend similar to the whole brain, where a decrease follows an initial increase in SampEn. The nMSE values are higher than those of edge-based nMSE across all scales in every RSN. The two-tailed paired t-test for the worst case is $t_{966}=13.7579$, $p<2\times 10^{-39}$ (uncorrected). The results also show that the networks have different levels of complexity when compared with the whole brain.

Figure 4 shows the variations of the mean (i.e., average of scale factors 1–10) MSE value of each RSN. There is a significant average difference between the mean nMSE and the mean edge-based nMSE values in every RSN. The two-tailed paired *t*-test for the worst case is $t_{966}=34.2972$, $p<10^{-169}$ (uncorrected), and the mean nMSE has more outliers than the mean edged-based nMSE. In addition, the mean nMSE has a smaller range, between the 25th and 75th percentile values. This suggests that the mean nMSE does not capture as much of the intrinsic patterns in subjects as the mean edge-based MSE does, but it captures much of the network differences. Another clear difference in most subjects is that the mean nMSE is higher than the mean edge-based nMSE in all RSNs. In short, the results from the network-level MSE analysis suggest that the inter-network differences are captured much better by the mean nMSE, while the intrinsic patterns in subjects are captured much better by the mean edge-based nMSE.

**Figure 5:**
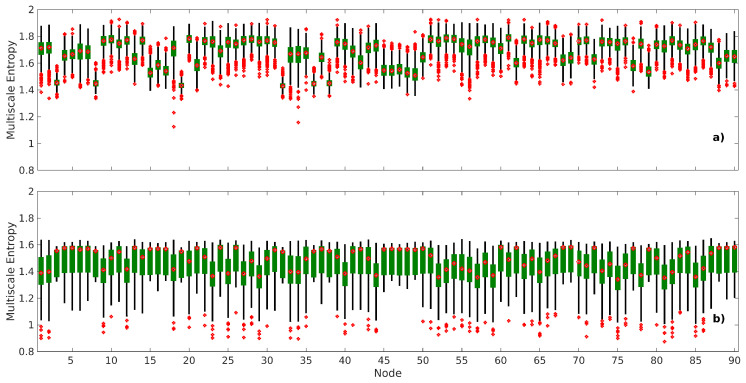
Box plots of the average MSE values of scale factors 1–10 for each node. (**a**) mean nMSE; and (**b**) mean edge-based nMSE. On each box, the central mark shows the median, bottom and top edges show the 25th and 75th percentiles, respectively, and the whiskers extend to the most extreme data points that are not considered as outliers, which are defined as 1.5 times the interquartile range away from the top or bottom of the box. Nodes belonging to the RSNs are shown in parentheses—auditory (1–3), basal ganglia (4–7), dorsal default mode network (8–17), primary visual (18–19), language (20–26), left executive control network (27–32), sensorimotor (33–38), posterior salience (39–50), precuneus (51–54), higher visual (55–56), right executive control network (57–62), anterior salience (63–69), ventral default mode network (70–79), visuospatial (80–90).

Figure 5 shows the node-level results (i.e., the variations of the mean MSE value of each node). Similar to the network-level case, there is a significant average difference between the mean nMSE and mean edge-based nMSE values in every node. The two-tailed paired t-test for the worst case is $t_{966}=4.6786$, $p<3\times 10^{-6}$ (uncorrected), and the spread of the mean nMSE data was smaller compared to the mean edge-based nMSE. These results are consistent with the network-level MSE analysis (see Figure 4) in that the mean edge-based nMSE is capturing much more intrinsic information compared to the mean nMSE, which captures the difference among networks. In addition, Figure 5 shows that the mean MSE of some intra-network nodes has different median values.

**Figure 6:**
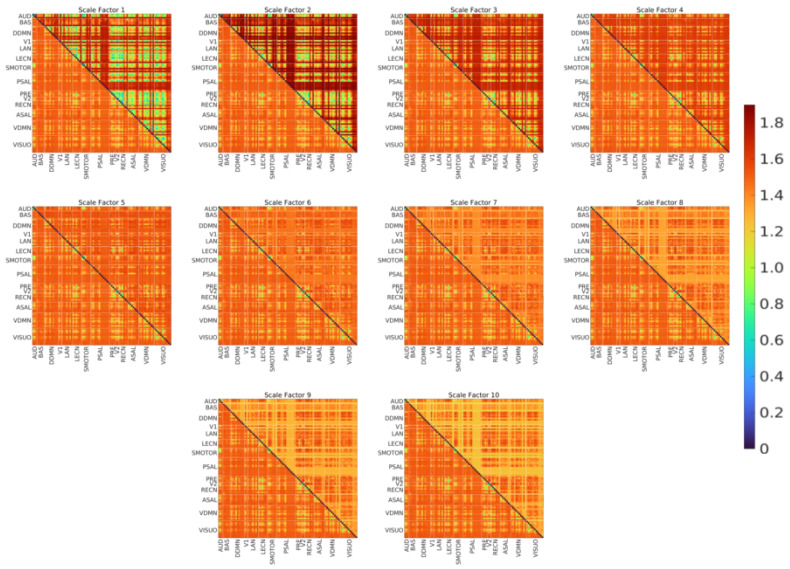
eMSE of edges. Upper triangular part—group mean eMSE for the noted scale factor, lower triangular part—grand mean eMSE (same in all matrices). The color bar on the right displays the mapping of the eMSE value to the color map. AUD-auditory, BAS-basal ganglia, DDMN-dorsal default mode network, V1-primary visual, LAN-language, LECN-left executive control network, SMOTOR-sensorimotor, PSAL-posterior salience, PRE-precuneus, V2-higher visual, RECN-right executive control network, ASAL-anterior salience, VDMN-ventral default mode network, VISUO-visuospatial.

**Figure 7:**
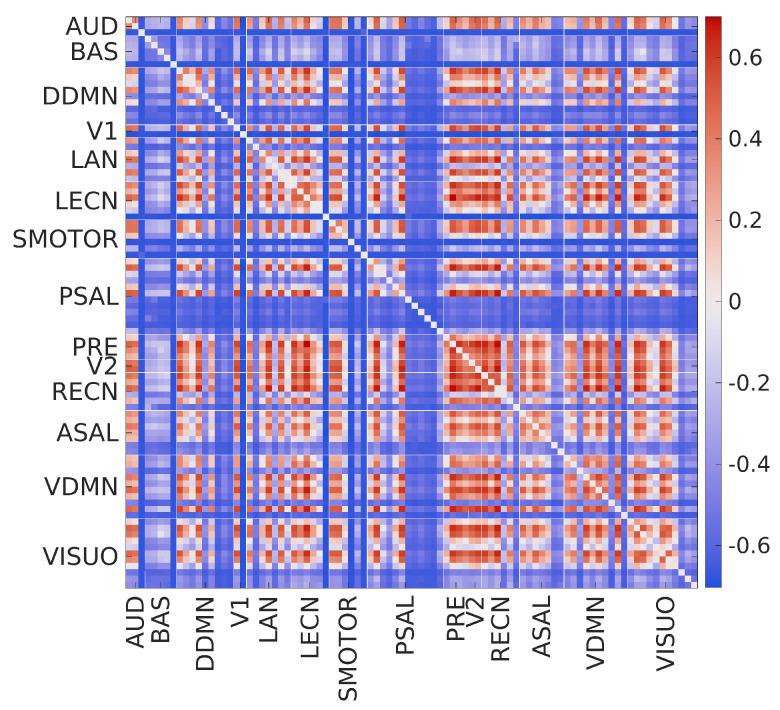
Divergence color map for the edges. The color bar on the right displays the mapping of the SampEn difference to the color map. AUD-auditory, BAS-basal ganglia, DDMN-dorsal default mode network, V1-primary visual, LAN-language, LECN-left executive control network, SMOTOR-sensorimotor, PSAL-posterior salience, PRE-precuneus, V2-higher visual, RECN-right executive control network, ASAL-anterior salience, VDMN-ventral default mode network, VISUO-visuospatial.

Figure 6 shows the edge-level results (i.e., the group mean eMSE of edges across scales). The upper triangular part of each matrix shows the group mean eMSE of edges for the respective scale, and the lower triangular part shows the grand mean (averaged over all subjects and scales) eMSE of edges. Figure 7 shows the divergence color map for the edges obtained using the difference between SampEn at scale ten and scale two. Note that scale two was chosen as the reference because this scale had the highest whole-brain entropy. Therefore, a positive value in the divergence color map represents an increase in SampEn and vice versa. The major observations from the edge-level results are as follows:

1. The group mean eMSE of most edges was highest for scale 2, consistent with the whole-brain (see Figure 2) and network-level (see Figure 3) results.
2. The matrix for scale 5 shows that the group mean eMSE values are most similar to the grand mean eMSE values.
3. There is a large variation in complexity among all edges at fine scales, which decreases at coarse scales.
4. The complexity of intra-network edges is lower than inter-network edges in many RSNs across scales. An example of this is the ventral default mode network (VDMN).
5. There is a large variation in complexity of all edges in many nodes in various RSNs at fine scales. An example of this is the dorsal default mode network (DDMN), and these variations decrease at coarse scales.
6. About 70% of edges show a decrease in SampEn as the scale increases. The remaining 30% show a diverging pattern with the SampEn increasing, which shows that the complexity is not correlated to the variance of coarse-grained signals [35].

Thus, additional information is obtained from an edge-level analysis.

### 2.3. Cognitive Behavioral Prediction Correlation Scores and Selected Features

The leave-one-subject-out SVR models that used the MSE of nodes and edges as features were able to predict individual fluid intelligence scores. Table 1 shows the prediction results for several combinations of pattern length and tolerance and the number of selected features. With m=3, r=0.2, and 50 features selected in the SVR training, the correlation of predicted and actual fluid intelligence scores using nMSE, edge-based nMSE, and eMSE features were 0.249, 0.202, and 0.240, respectively. The permutation test resulted in a *p*-value < 0.001 with 1000 permutations; therefore, the correlation results are statistically significant. Note that when 900 nMSE and edge-based nMSE features were selected, all 90 nodes and 10 scale factors were used to predict fluid intelligence. However, 900 selected features using eMSE represented only 2.25% of the 4005 edges and 10 scale factors. Although no attempt was made to find an optimal number of features to include in the SVR models, it is clear that including a large number of features does not improve the prediction accuracy. Surprisingly, a small number of features was sufficient to obtain the best or second-best correlation value for the cases that were studied.

**Figure 8:**
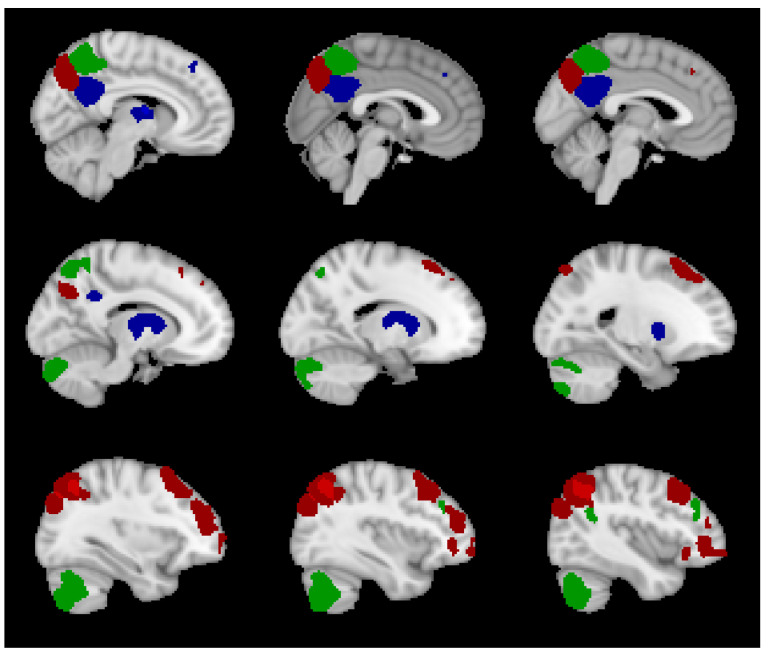
Sagittal views of brain regions selected in predicting fluid intelligence. Red—common regions selected when using nMSE and edge-based nMSE features, blue—regions selected with only nMSE features, green—regions selected with only edge-based nMSE features.

Further analysis was carried out to identify the most significant node and edge features involved in predicting fluid intelligence when 50 features were selected. Table A1 shows the nodes and the RSNs that the nodes were part of when nMSE and edge-based nMSE were used to predict fluid intelligence. While several nodes in different RSNs were involved, the most significant number of nodes came from the RECN and the left executive control network (LECN), followed by the language and precuneus networks. Although nodes selected with nMSE were mostly from scales 1 and 2, others were selected from scales 5, 7, 9, and 10. On the other hand, with edge-based nMSE, nodes were selected only from scales 1–3. Many nodes selected by both nMSE and edge-based nMSE were the same. Figure 8 shows the sagittal views of these common brain regions, as well as those that were specific to each complexity method.

**Figure 9:**
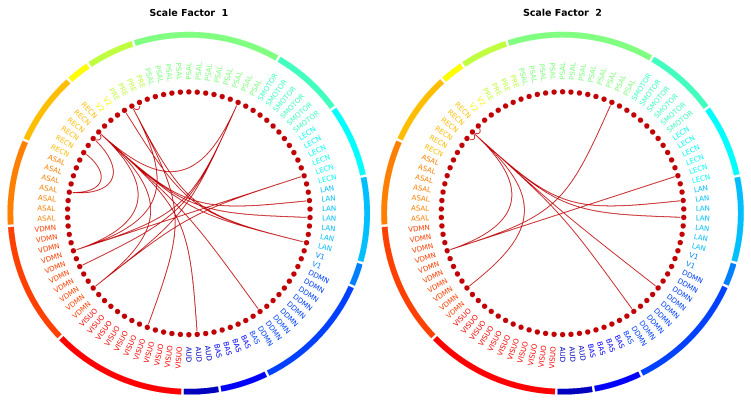
Significant edges in predicting fluid intelligence using eMSE features. Connectogram for scale factor 3 not shown because it had only one edge with an intra-node connection in the RECN. AUD-auditory, BAS-basal ganglia, DDMN-dorsal default mode network, V1-primary visual, LAN-language, LECN-left executive control network, SMOTOR-sensorimotor, PSAL-posterior salience, PRE-precuneus, V2-higher visual, RECN-right executive control network, ASAL-anterior salience, VDMN-ventral default mode network, VISUO-visuospatial.

Similar to the edge-based nMSE, features selected with eMSE came from scales 1–3. Figure 9 shows these edges for scales 1 and 2. The figure does not include scale 3 because there was only one intra-network edge between node #58 (right middle frontal gyrus) and node #59 (right inferior parietal gyrus, supramarginal gyrus and angular gyrus) in the RECN network. The edges were predominantly inter-network edges between the RECN and the VDMN, and a few intra-network edges were found in the RECN and precuneus.

**Figure A1:**
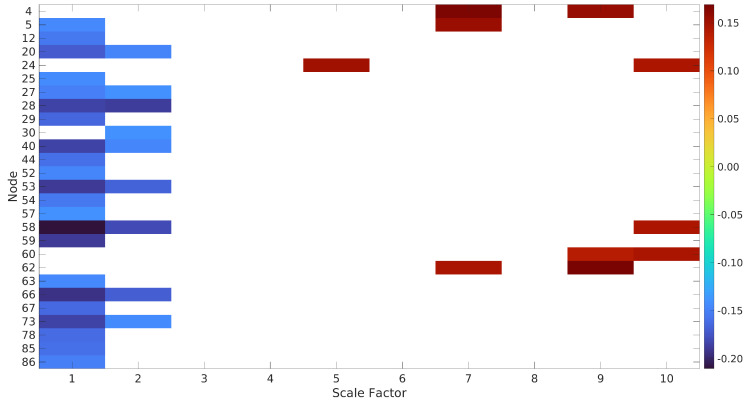
Group mean correlation of selected nMSE features with fluid intelligence across scale factors. The color bar on the right displays the mapping of the correlation value to the color map.

Figure A1 shows the group mean correlation values of the selected nMSE features, which were common in all subjects to predict fluid intelligence in the leave-one-subject-out SVR models. The nMSE values were negatively correlated with fluid intelligence scores at fine scales and positively correlated at coarse scales. On the other hand, selected eMSE features had all negative correlations. However, it should be noted that the eMSE features had both negative and positive correlation values across scales; those selected to predict fluid intelligence were all negatively correlated.

## 3. Discussion

This study is the first to consider MSE of functional complexity using dFC obtained from the BOLD PCC, and expands brain complexity analysis to the edge level. In addition, neuronal and functional complexities estimated using MSE were compared at different levels of the brain’s organization. Furthermore, neuronal and functional complexities were used to predict fluid intelligence. A large number of subjects (n = 967) with high temporal resolution data and long rfMRI data sets was studied here.

The brain complexity at the whole-brain level estimated using nMSE and eMSE was different because nMSE captures neuronal complexity from the BOLD time series, while eMSE captures functional complexity from the dFC edge time series. The decrease in neuronal complexity at higher scales was similar to some previous findings [24,36], but is different from [34], which may be due to differences in the number of subjects included in the study, the length of time series data, and the use of the parcellation scheme. However, the initial increase followed by a decrease in functional complexity when the scale is increased is similar to the “skewed inverted-U pattern” in [34]. The network-level complexity analysis reveals that the neuronal and functional complexities differ among networks. Except for the precuneus, higher visual, and RECN, all networks had trends similar to the whole-brain complexity. The results showed that some information is lost in averaging the SampEn due to mixing of complexity measures [34] because MSE trends were not the same in every RSN as in the whole brain. The edge-level results show that additional information is obtained compared to those from the node- and network-level analyses. A previous study with windowed dFC had shown that there exists a difference in SampEn measured throughout the brain at different organization levels [29].

The abbreviated version of Raven’s Progressive Matrices would be considered as a “fair” quality measure to assess fluid intelligence, with the expected correlation in the range 0.50 to 0.71 [37]. The fair quality measure in itself may lead to a lower correlation between the fluid intelligence and the BOLD time series data. Moreover, a correlation of 0.2 is considered “typical” in research related to studying individual differences [38]. Considering the large number of subjects, the correlation results for predicting fluid intelligence were well within the range of some published results [10,39,40]. Although from an absolute perspective the correlation would be considered as small, valuable insights were obtained on the most significant features selected with different complexity levels (See Table A1 and Figure 9).

The brain regions in the RECN and LECN, such as frontal gyrus, angular gyrus, and parietal gyrus were selected as significant features in the leave-one-subject-out SVR models. These regions are known to be influential on fluid intelligence [33,39,41]. In addition, fluid intelligence is related to activity in the bilateral superior, inferior, and middle frontal gyri, as well as the anterior cingulate and paracingulate cortex [41]. The selected features corroborate with these past studies. The group mean correlation of nMSE with fluid intelligence was negative at fine scales and positive at coarse scales. Recent studies have shown the FC and SampEn have a similar correlation trend [34,36,42,43], and FC had a positive correlation with fluid intelligence [10,39,41,44]. In addition, studies have shown that fluid intelligence depends on synchrony in the brain’s intrinsic network [45]. Higher intelligence was shown to be associated with the brain’s capacity to adapt to a new state during the task with small changes in FC [44]. The synchronization and smaller changes in network dynamics are related to decreasing SampEn at fine scales [34,36], which is related to well-organized brain network dynamics.

Selecting appropriate MSE parameters is crucial to accurately estimate brain complexity. The relative error method [20,21] can be successfully implemented to find parameter combinations that satisfy some user-defined optimization criterion. Here, it can be seen that the relative error was well below the selected threshold of 0.1, and potential combinations with a relative error of less than 0.05 were present, which is the best value for the efficiency metric reported in [20]. The relative error was small compared to some recent studies [13,21], which may be due to the high quality of data used, high temporal resolution, and long rfMRI data sets.

### Limitations and Future Direction

The results presented in this study, which considered a large number of healthy young adults, provide a baseline for other studies that explore the use of MSE to examine changes in brain function related to aging, diseases, and clinical disorders. Further studies are needed to ascertain if functional complexity provides a better differentiation ability than neuronal complexity by comparing between cohorts with diseases or neurological impairments and healthy control cohorts. Although many common regions were identified to be significantly correlated to fluid intelligence by considering neuronal and functional complexities, there were other areas specific to the two types of complexities. With neuronal complexity, features were selected from a wide range of scales. On the other hand, features were selected from the first three scales with functional complexity, which is advantageous as it precludes the need for long BOLD signals to calculate good estimates of MSE. Future work is required to determine if only the first few scales are sufficient for estimating brain complexity. In addition, advanced nonlinear mathematical models are needed to predict cognitive performance with higher accuracy using the BOLD time series data.

Another limitation of this study is that MSE was estimated in a traditional manner by considering the tolerance *r* to be scale-invariant. A number of modifications and alternate coarse-graining procedures have been proposed to improve the accuracy of estimating MSE and mitigate the limitation of using scale-invariant tolerance values. See [46] for a review of these refined methods. More recently, a theoretical approach was presented to analytically calculate a new MSE measure using state-space models [47,48,49]. In addition, the importance of considering signal normalization and spectral content was shown using simulated and empirical data [50]. Recommendations were made for the steps to be followed to safeguard against biases in traditional MSE implementations. A comparison of the results presented in this study, including the diverging patterns shown in Figure 3 and Figure 7, with recently published refined methods being of great interest; they are recommended for future studies.

## 4. Materials and Methods

### 4.1. fMRI Data

The data was acquired with multiband echo-planar imaging at a temporal resolution (TR) of 0.72 s per volume and 2-mm isotropic voxels for about 1200 young adults (ages 22–35) from families with twins and non-twins using a 3T imaging scanner at Washington University in St. Louis. The 3T rfMRI data was acquired in four runs of approximately 15 min each, two runs with right-to-left and left-to-right phase encoding protocols on day 1 and two similar runs on day 2 were used in this study. To remove the effects of structured artifacts, the data have been run through HCP FIX-ICA denoising. A detailed description of the minimal preprocessing steps applied to the data can be found in [1,51], and a description of the FIX approach in [52].

Some subjects in the HCP data set were excluded for the following reasons.

1. Missing rfMRI sessions—174 subjects were missing in one of the resting-state sessions.
2. High average framewise displacement [53]—16 subjects had an average framewise displacement greater than four standard deviations of the group, which introduces head motion artifacts.
3. Missing time series data—six subjects had less than 1200 points in one of the sessions.
4. Misalignment—one subject was misaligned in standard space and was missing some brain regions.
5. Low Mini-Mental Status Exam (MMSE) score [54]—33 subjects had an MMSE score of 26 or lower, which can be an indicator of cognitive impairment.
6. Missing fluid intelligence score—nine subjects were missing the fluid intelligence score.

The 967 subjects (451 males and 516 females) that remained after applying the exclusion criteria were included in this study.

Brain parcellation was performed using an atlas with 90 functional brain regions of interest, also known as nodes, defined across 14 major RSNs (https://findlab.stanford.edu/functional_ROIs.html). Time series of the 90 nodes, which were obtained using MATLAB based SPM (https://www.fil.ion.ucl.ac.uk/spm/) and REX toolbox (https://web.mit.edu/swg/rex/rex.m) from the four sessions, were detrended [MATLAB-detrend] and z-score normalized [MATLAB-zscore] before being concatenated to yield a total of 4800 data points. The time series were not filtered because studies have shown that useful neuronal related signals are present at higher frequencies up to 0.5 Hz [1,55,56]. Note that for clarity and completeness, the software package and commands, with any options, that were used to obtain the results are included within square brackets throughout the manuscript. MATLAB 2018a and SPM12 were used to obtain the results presented here.

### 4.2. Node and Edge Complexities

Brain complexity was estimated in two steps using MSE. First, the multiple coarse-grained time series of length (N/l) was formed by averaging consecutive, non-overlapping data points of increasing length. Second, the SampEn of each coarse-grained time series was calculated (WFDB Toolbox for MATLAB—https://physionet.org/physiotools/matlab/wfdb-app-matlab/). Three parameters must be defined: (i) pattern length *m*—the number of data points for pattern matching; (ii) tolerance (similarity factor) *r*—the fraction of standard deviation of the time series; and (iii) scale factor *l*—the scale factor of coarse-graining.

The time series of the 90 nodes, each having 4800, time points were used to calculate the nMSE values. On the other hand, the eMSE values were calculated using PCC [57], which yields dynamic connectivity matrices of size 90×90 at every time point. The phase of the time series data for the *i*th node, θ(i,t), was first calculated using Hilbert transform [MATLAB-hilbert,angle]. Next, the phase value was used to calculate the dFC matrix at time *t* using phase coherence between brain regions *i* and *j* as [57]:

(1) dFC(i,j,t)=cos(θ(i,t)−θ(j,t))

where cos() is the cosine function. Because the dFC matrices are symmetric, only the upper triangular part was used to obtain the edge connectivity changes with time. This yielded the time series for 4005 edges with their connectivity variations along 4800 time points, and eMSE was calculated for these edges. For any given time series {$x_{i}\;,\;i=1,2,3,\cdots ,N$}, the course-grained time series, $y^{l}$, and SampEn are calculated as follows:

(2) $$y_{j}^{l}=\frac{1}{l}\sum_{i=(j-1)l+1}^{jl}x_{i},\;1\leq j\leq \frac{N}{l}$$

(3) $$SampEn\left(m,r,N\right)=-ln\frac{P_{m+1}\left(r\right)}{P_{m}\left(r\right)}$$

where *P* is the probability that the time points are within tolerance *r*.

### 4.3. Optimal Parameter Selection for Calculating SampEn of BOLD Signals

Selecting the optimal combination of MSE parameters *m*, *r* and *l* is a challenging task. The relative error method presented in [21] was used in this study to find the acceptable MSE parameter space. Using the time series of all CSF signals, the relative error in SampEn estimation was calculated as:

(4) $$RE=1.96\times \frac{\frac{\sigma_{SampEn}}{\mu_{SampEn}}}{2}$$

where $\mu_{SampEn}$ and $\sigma_{SampEn}$ are the mean and standard deviation of the SampEn of CSF signals, respectively. The acceptable MSE parameter space of BOLD signals was determined by using a threshold value of 0.1 for RE [13,21] and excluding parameter combinations that failed to calculate SampEn for a subject using CSF signals.

### 4.4. Cognitive Behavioral Prediction

Fluid intelligence, which is related to the intrinsic cognitive ability that is correlated with reasoning and problem solving irrespective of acquired knowledge [39], was used to evaluate the significance of neuronal and functional complexities. A conservative leave-one-subject-out cross-validation method with support vector regression [MATLAB - fitrsvm] was used to predict individual fluid intelligence scores. These scores were measured in the HCP using Form A of an abbreviated version of Raven’s Progressive Matrices [32]. Three different cases were studied where features were selected to predict fluid intelligence using nMSE, edge-based nMSE, and eMSE values across multiple scales.

**Figure 10:**
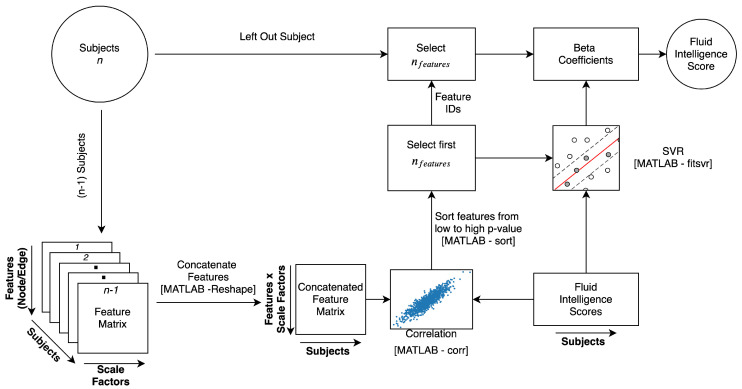
Schematic of feature selection and fluid intelligence prediction.

Figure 10 shows the four main steps for identifying the features and predicting fluid intelligence. First, the features (MSE values) across multiple scales were concatenated for each subject. For the nMSE and edge-based nMSE cases, the (90 ×*l*) matrix for each subject resulted in a column vector with dimension 90 *l*. In the higher dimensional eMSE case, the (4005 ×*l*) matrix for each subject resulted in a column vector with dimension 4005 *l*. Second, the correlation between the features and fluid intelligence was calculated. Third, the features were sorted from the lowest to the highest *p*-value of correlation, and the first *n* features were selected [39,58] for use in the SVR model. No correction for multiple comparisons was made since the feature selection method has a built-in guard against false positives, and the model fails to generalize for independent data when the false-positive proportion is higher in feature selection [39]. Finally, the selected features were then used to learn the SVR models, which were in turn used to predict the fluid intelligence scores of the left-out subjects. Because the choice of the number of features to be selected was arbitrary, a range of numbers was explored. After the leave-one-subject-out trials were completed, the prediction performance was calculated by correlating the predicted and actual fluid intelligence scores. The raw fluid intelligence scores were used here, and no control for confounding effects of age, gender, motion, and family was considered.

The statistical significance of the leave-one-subject-out method was assessed using permutation testing [10,58,59], which verifies whether the final prediction correlation was significantly better than the results expected by chance. Keeping feature selection and SVR training steps the same, the fluid intelligence scores of subjects were permuted 1000 times, and the correlation between predicted and actual fluid intelligence scores were re-calculated. The *p*-value then represents the probability of observing the reported accuracy by chance.

## Figures and Tables

**Table 1:** Correlation of predicted and actual fluid intelligence scores. The results for the case described in Section 2.3 are highlighted in bold italic font.

Complexity Level	Pattern Length, m	Tolerance, r	Number of Selected Features			
			25	50	100	900
**nMSE**	1	0.05	0.168	0.174	0.134	0.178
	2	0.10	0.231	0.179	0.200	0.150
	3	0.20	0.200	***0.249***	0.133	0.158
**Edge-based** **nMSE**	1	0.05	0.079	0.239	0.319	0.237
	2	0.10	0.159	0.152	0.246	0.181
	3	0.20	0.178	***0.202***	0.195	0.207
**eMSE**	1	0.05	0.180	0.127	0.171	0.165
	2	0.10	0.160	0.238	0.210	0.126
	3	0.20	0.237	***0.240***	0.234	0.165

## Abbreviations

The following abbreviations are used in this manuscript:

ASAL	Anterior salience network
AUD	Auditory network
BAS	Basal ganglia network
BOLD	Blood oxygen-level dependent
CSF	Cerebrospinal fluid
DDMN	Dorsal default mode network
dFC	Dynamic functional connectivity
eMSE	Edge multiscale entropy
HCP	Human Connectome Project
LAN	Language network
LECN	Left executive control network
MSE	Multiscale entropy
nMSE	Node multiscale entropy
PCC	Phase coherence connectivity
PRE	Precuneus network
PSAL	Posterior salience network
rfMRI	Resting-state functional magnetic resonance imaging
RECN	Right executive control network
RSN	Resting-state networks
SampEn	Sample entropy
SMOTOR	Sensorimotor network
SVR	Support vector regression
V1	Primary visual network
V2	Higher visual network
VDMN	Ventral default mode network
VISUO	Visuospatial network

## Appendix A. Predicting Fluid Intelligence

Table A1 shows the selected nodes that were common in all subjects when predicting fluid intelligence using nMSE and edge-based nMSE features. Although 50 nodes were selected for leave-one-subject-out SVR training, the total number of nodes listed in the table are less than 50 because a few nodes differed in the leave-one-subject-out trials. Figure A1 shows the group mean correlation of selected nMSE features with fluid intelligence across scales.

**Table A1:** Common nodes from all leave-one-subject-out trials for predicting fluid intelligence with m=3, r=0.2, and number of features = 50.

Network	Node Number	Brain Region	Scale Factor	
			nMSE	Edge-Based nMSE
**BAS**	4	Left Thalamus, Caudate	7, 9	
	5	Right Thalamus, Caudate, Putamen	1,7	
**DDMN**	12	Posterior Cingulate Cortex, Precuneus	1	
**LAN**	20	Inferior Frontal Gyrus	1, 2	1, 2
	22	Left Middle Temporal Gyrus, Angular Gyrus		1
	23	Left Middle Temporal Gyrus, Superior Temporal Gyrus, Supramarginal Gyrus, Angular Gyrus		1
	24	Right Inferior Frontal Gyrus	5, 10	
	25	Right Supramarginal Gyrus, Superior Temporal Gyrus, Middle Temporal Gyrus	1	1
**LECN**	27	Left Middle Frontal Gyrus, Superior Frontal Gyrus	1, 2	1, 2
	28	Left Inferior Frontal Gyrus, Orbitofrontal Gyrus	1, 2	1, 2, 3
	29	Left Superior Parietal Gyrus, Inferior Parietal Gyrus, Precuneus, Angular Gyrus	1	1, 2
	30	Left Inferior Temporal Gyrus, Middle Temporal Gyrus	2	1, 2
**PSAL**	39	Left Middle Frontal Gyrus		1, 2
	40	Left Supramarginal Gyrus, Inferior Parietal Gyrus	1, 2	1, 2
	44	Right Supramarginal Gyrus, Inferior Parietal Gyrus	1	1
**PRE**	52	Precuneus	1	1
	53	Left Angular Gyrus	1, 2	1, 2, 3
	54	Right Angular Gyrus	1	1, 2
**RECN**	57	Right Middle Frontal Gyrus, Right Superior Frontal Gyrus	1	1, 2
	58	Right Middle Frontal Gyrus	1, 2, 10	1, 2, 3
	59	Right Inferior Parietal Gyrus, Supramarginal Gyrus, Angular Gyrus	1	1, 2
	60	Right Superior Frontal Gyrus	9, 10	
	61	Left Crus 1, Crus II, Lobule VI		1, 2
	62	Right Caudate	7, 9	1
**ASAL**	63	Left Middle Frontal Gyrus	1	
	66	Right Middle Frontal Gyrus	1, 2	1, 2
	67	Right Insula	1	
**VDMN**	73	Left Middle Occipital Gyrus	1, 2	1, 2
	75	Precuneus		1
	78	Right Angular Gyrus, Middle Occipital Gyrus	1	1, 2
**VISUO**	85	Right Inferior Parietal Lobule	1	1
	86	Right Frontal Operculum. Inferior Frontal Gyrus	1	1
